# Cisplatin-Induced Ototoxicity: Effects, Mechanisms and Protection Strategies

**DOI:** 10.3390/toxics3030268

**Published:** 2015-07-15

**Authors:** Angela Callejo, Lara Sedó-Cabezón, Ivan Domènech Juan, Jordi Llorens

**Affiliations:** 1 Unitat Funcional d’Otorrinolaringologia i Al·lèrgia, Institut Universtiari Quirón Dexeus, 08028 Barcelona, Catalonia, Spain; E-Mail: 35019idj@gmail.com; 2 Departament de Ciències Fisiològiques II, Universitat de Barcelona, 08907 L’Hospitalet de Llobregat, Catalonia, Spain; E-Mails: larasedo@hotmail.es (L.S.-C.); jllorens@ub.edu (J.L.); 3 Servei d’Otorrinolaringologia, Hospital Universitario de Bellvitge, 08907 L’Hospitalet de Llobregat, Catalonia, Spain; 4 Institut d’Investigació Biomèdica de Bellvitge (IDIBELL), 08907 L’Hospitalet de Llobregat, Catalonia, Spain

**Keywords:** cisplatin, ototoxicity, hearing loss, protective treatment, intratympanic

## Abstract

Cisplatin is a highly effective chemotherapeutic agent that is widely used to treat solid organ malignancies. However, serious side effects have been associated with its use, such as bilateral, progressive, irreversible, dose-dependent neurosensory hearing loss. Current evidence indicates that cisplatin triggers the production of reactive oxygen species in target tissues in the inner ear. A variety of agents that protect against cisplatin-induced ototoxicity have been successfully tested in cell culture and animal models. However, many of them interfere with the therapeutic effect of cisplatin, and therefore are not suitable for systemic administration in clinical practice. Consequently, local administration strategies, namely intratympanic administration, have been developed to achieve otoprotection, without reducing the antitumoral effect of cisplatin. While a considerable amount of pre-clinical information is available, clinical data on treatments to prevent cisplatin ototoxicity are only just beginning to appear. This review summarizes clinical and experimental studies of cisplatin ototoxicity, and focuses on understanding its toxicity mechanisms, clinical repercussions and prevention strategies.

## 1. Introduction

Cisplatin (*cis*-diamminedichloroplatinum II) (CDDP) is a highly effective chemotherapeutic agent used to treat solid tumors, including ovary, testis, bladder, non-small cell lung, and head and neck solid tumors [1,2,3]. The mechanism accounting for its antitumor activity is the formation of covalent bonds between the cisplatin platinum (Pt) atom and DNA purine bases in the N7 position, which generate intra- and inter-strand cross-links that have been demonstrated *in vitro* to trigger apoptotic and necrotic cell death [4]. The main reported side effects associated with its use are nephrotoxicity, peripheral neurotoxicity and ototoxicity [1,2]. While nephrotoxicity can be ameliorated by increasing saline hydratation as well as mannitol diuresis, there are no approved preventive treatments available for ototoxicity and neurotoxicity. Despite the indolence of ototoxicity in comparison to other side effects, it has attracted much interest due to the high rate of affected patients, a fact that makes it the most common dose-limiting side effect [1,3,5,6,7]. Numerous clinical and experimental studies have attempted to elucidate the cisplatin mechanism of otoxicity, clinical implications and prevention strategies.

## 2. Mechanism of Ototoxic Action of Cisplatin

Recent studies pointed out that the cytostatic/cytotoxic effects of CDDP originate from both nuclear and cytoplasmic signaling pathways [8]. It is known that only ~1% of intracellular CDDP forms covalent bonds with nuclear DNA [9], but CDDP also exerts prominent cytotoxic effects in enucleated cells (cytoplasts) [10]. The exact molecular mechanisms that explain cytotoxic potential of cytoplasmic CDDP are not yet known, but they are believed to involve: (1) the accumulation of reactive oxygen species (ROS) and nitric oxide (NO) which exert direct cytotoxic effects by favoring the opening of the permeability transition pore complex (PTPC) [11]; (2) the induction of a mithocondrial outer membrane permeabilization (MOMP)-stimulatory signal via BAK1 (pro-apoptotic BCL-2 family member), VDAC1 (PTPC component voltage-dependent anion channel1) and BAX (BAK1 homolog) [12]; and (3) the activation of a cytosplasmic pool of p53, which is capable to promote the MOMP via [13]. 

Transport of cisplatin and its metabolites across the cell membrane, from the blood to the cytosol, is accomplished by a number of membrane transporters. The mammalian copper transporter 1 (CTR1) or solute carrier 31A1 (SLC31A1) is a membrane protein that plays a significant role in the cellular cisplatin uptake [14]. CTR1 carries out a vital physiological function supplying the cell with copper, which is an essential cellular nutrient used in a broad range of enzymatic reactions. The natural substrate of CTR1 is monovalent copper (Cu^+^). Copper ions bind to methionine-rich motifs of the extracellular domain of CTR1 [15] and trigger transporter internalization. Cisplatin seems to bind to the same extracellular methionine-rich domain where Cu^+^ does, allowing the entrance of cisplatin into the cell [15]. Since several cell lines from human tumor samples express CTR1-mRNA, this transporter might be the uptake route of cisplatin in cancer cells [16]. Indeed, high expression levels of CTR1 have been associated with cisplatin therapeutic success [17] and CTR1 mutations are associated with cisplatin resistance [18]. Copper transporter 2 (CTR2), also called SLC31A2, is a membrane protein with structural homology to CTR1, mainly expressed in late endosomes and lysosomes [19], where it mediates the efflux of copper under conditions of low environmental copper concentration [20]. High expression of CTR2 seems to be associated with resistance to cisplatin [21], while decreased CTR2 expression has been associated with an increased cisplatin cytotoxicity [19,21]. Copper-transporting ATPases, ATP7A and ATP7B, play an important role in regulating the cellular copper levels, mediating its efflux from the cell or its distribution to specific sub-cellular compartments [22,23,24,25], to avoid too high intracellular copper concentrations, which might be toxic. Organic cation transporters (OCT1–3) or SLC22A1–3 are membrane proteins that specifically interact with cisplatin [26] and are highly expressed in excretory organs such as the liver and the kidneys [27]. However, OCT2 has been demonstrated to be also expressed in hair cells of the organ of Corti and in the cells of the stria vascularis of the cochlea in mice [28]. The OCT-2 non-synonymous single nucleotide polymorphism (SNP) 808 G/T has been associated with protection from cisplatin-induced nephrotoxicity [29] and ototoxicity in pediatric population [30]. Multidrug and Toxin Extrusion Protein 1 (MATE1) also called SLC47A1 is a cisplatin transporter involved in its efflux from renal proximal tubule cells in humans to the urine, and related to its nephrotoxicity [31]; up to now, no association with ototoxicity has been described. 

CDDP is chemically inert until one or both of its *cis* chloro groups are replaced by water molecules [32]. Such aquation reaction takes places in lower proportion in the blood, but mostly occurs spontaneously in the cytoplasm, due to the relative low concentration of chloride ions (~2–10 mM, as compared with ~120 mM in the extracellular space) [33,34,35]. Once inside the cell, *cis*-diammine(aqua)chloroplatinum (II) (or monohydrate cisplatin complex) is capable of causing death by at least two different mechanisms: one dependent on p53 and caspases [36,37,38], and one mediated by protein kinases [39]. Both mechanisms have been demonstrated *in vivo* in rats [40,41]. 

In p53 and caspase-dependent apoptosis, exposure to high doses of cisplatin inhibits the activity of some antioxidant enzymes (superoxide dismutase, catalase, glutathione peroxidase and glutathione reductase) and stimulates the activity of others, such as NOX-3 (a nicotinamide adenine dinucleotide phosphate (NADPH)-oxidase isoform that is highly expressed in the cochlea), with a consequent increase in lipid peroxidation, causing high levels of reactive oxygen species (ROS) [42,43]. The superoxide radicals thus generated can develop four cytotoxic actions: (a) interact with nitric oxide (NO) and form peroxynitrite which will nitrosylate proteins, inactivating them [44]; (b) form hydroxyl free radicals that, after interacting with iron (Fe), can react with polyunsaturated fatty acids (PUFA) of the cell membrane lipid bilayer, generating the reactive aldehyde 4-hydroxynonenal (4-HNE) [44] that triggers calcium influx into the cell, leading to apoptosis [42,45]; (c) inactivate antioxidant enzymes [46]; and (d) trigger cytosolic migration of Bax, with consequent activation of the mitochondrial apoptosis signaling pathway, which involves the release of cytochrome c from mitochondria and the activation of caspases 9 and 3 [37,38].

In addition to p53 and caspase-dependent apoptosis, cells exposed to cisplatin show overexpression of the extracellular regulatory kinase (ERK) 1/2 and nuclear factor κB (NFκB), which stimulate proinflammatory cytokines, such as tumor necrosis factor α (TNF-α), interleukin (IL)-1β and IL-6. This causes nuclear fragmentation, rearrangement of the actin cytoskeleton and cell death [39,41].

The cochlea is a highly complex organ comprised of many cell types whose susceptibility to the cytotoxic mechanisms of cisplatin varies. Several studies have addressed mechanisms of cisplatin toxicity that operate in particular cells or tissues of the cochlea.

In type I fibrocytes from the sidewall of the spiral ligament, cisplatin activates big conductance potassium channels. The increased potassium efflux causes a loss of intracellular osmotic pressure and ionic strength. This disrupts the electrochemical gradient of the endolymph, where the concentration of potassium is high and that of calcium is low compared to the surrounding perilymph [47]. These alterations in ion balance trigger pro-apoptotic pathways, leading to cell death [48,49]. 

In the stria vascularis and spiral ligament of the mouse cochlea, induction of nuclear-factor kappa B (NFκB) and inducible nitric oxide synthase (iNOS) have been demonstrated after systemic exposure to cisplatin, which suggests that nitric oxide (NO) plays a significant role in mediating cisplatin’s ototoxicity [50]. Studies in rats treated with cisplatin showed evidence of NFκB activation in the organ of Corti, the spiral ligament and the stria vascularis [41]. The proapoptotic role of NFκB was demonstrated in the auditory cell line HEI-OC1 by the use of NFκB inhibitors, which decreased the activation of caspase 3 resulting from cisplatin exposure [51]. Studies *in vitro* with HEI-OC1 cells and *in vivo* in mice have also demonstrated a cytotoxic role of proinflammatory cytokines such as TNF-alpha, IL-1beta and IL-6 in cisplatin ototoxicity [52].

Cisplatin may also act directly on the DNA, particularly binding to nucleophilic N7 sites on purines, and cause cross-linking and adduct formation. The resulting DNA damage is repaired by a system called “nucleotide excision repair” (NER), as described in previous studies on the use of cisplatin in the treatment of ovarian cancer and small-cell lung cancer [53,54,55]. This endogenous DNA repairing system can detect DNA lesions in two ways: global genome-NER (GG-NER) repairs DNA lesions in non-transcribed genes [56,57], which are identified by several proteins including the Xeroderma pigmentosum complementation group C protein (XPC) [58,59]; and transition coupled NER (TC-NER) repairs DNA lesions in transcribed genes and involves the activity of the Xeroderma pigmentosum complementation group A protein (XPA) [60]. Both XPC and XPA are essential for the proper function of the NER DNA repair system. Cytoplasm to nucleus translocation of both systems, XPA and XPC, has been shown in the cochleae of rats treated with cisplatin [61]. 

Cisplatin toxicity may also be counteracted by a number of cochlear endogenous defense systems, including: antioxidant enzymes, adenosine receptors, kidney injury molecule-1 (KIM-1), heat shock proteins, and heme oxygenase-1 [1]. Antioxidant enzymes (superoxide dismutase [SOD], catalase [CAT], glutathion reductase [GSH-R], GSH peroxidase [GSH-PX], glutathione-S-transferase and glutamyl cysteine synthetase) have a cytoprotective effect against cisplatin in *in vitro* models [1]; downregulation of these enzymes has been associated with increased levels of both malondialdehyde (a lipid peroxidation indicator) [62] and oxidized glutathione [63]. Activation of adenosine A1 receptors by exogenous agonists causes an increase in the expression of antioxidant enzymes, and the presence of adenosine A1 receptors in the cochlea has been demonstrated. Therefore, it is alleged that the activation of these receptors by endogenous molecules can contribute to the elimination of ROS generated by cisplatin [64]. In the proximal renal tubule epithelium, cisplatin exposure increases the expression of KIM-1, which has a repairing action on damaged renal tissue. *In vivo* and *in vitro* studies in the rat have subsequently shown that cochlear epithelium also overexpresses KIM-1 after a cisplatin insult [40]. Heat shock proteins (HSP-70, HSP-90, HSP-27) inhibit apoptosis in two different ways (inhibition of apoptosome assembly and inhibition of Jun *N*-terminal kinase [JNK]), as shown in *in vitro* studies using mouse utricular cells [65]. Heme oxygenase-1 (HO-1) is an anti-apoptotic gene, whose action is mediated by degradation products of the heme group: carbon monoxide, bilirubin and ferrous ion (Fe^2+^); all of which reduce the formation of ROS products [66]. The protective effect of the aforementioned cytoprotective endogenous molecules against cisplatin ototoxicity is limited, and cell death occurs when they are overcome [67]. 

## 3. Structural Lesions due to Cisplatin Ototoxicity

A number of experimental studies have described the structural damage caused by cisplatin and its metabolites in the cochlear cells of laboratory animals. The major observation is apoptotic degeneration of the hair cells in the organ of Corti. Fragmentation of double stranded DNA and other hallmarks of apoptosis have been observed to start in the outermost row of outer hair cells and then progress towards the innermost rows. Nevertheless, lesions in other structures have also been reported, including dehiscence of the myelin sheaths of type I neurons of the spiral ganglion, and depletion of the number of cytoplasmic organelles, edema, and cell shrinkage and lysis in the stria vascularis [68,69,70,71].

Topographic studies have demonstrated the existence of a gradient of lesion progression, so damage to the base of the cochlea is more severe than damage to the apex [1,2,3,6,72]. This explains why high frequencies are more affected or affected earlier than lower frequencies in the audiometric studies.

## 4. Functional Changes due to Cisplatin Ototoxicity

### 4.1. Auditory Functional Changes on Experimental Animals

The abovementioned structural lesions result in impaired cochlear function, as demonstrated in several experimental studies in guinea pigs, rats and gerbils: reduction of endocochlear potential (EP), elevation of compound action potential (CAP) and cochlear microphonic potential (CM) thresholds, reduced distortion product otoacoustic emissions (DPOAE), and increased thresholds of auditory evoked brainstem responses (ABR). A greater effect on high rather than low frequencies is a consistent finding [1].

The functional data are also consistent with histological evidence of the unique susceptibility of the outer hair cells to the action of cisplatin, both in humans and in experimental animals. Since DPOAE result from the activity of outer hair cells, and provide a quick, non-invasive and sensitive technique for evaluating the functional state of these cells, their study is useful for the evaluation of the experimental ototoxicity of cisplatin, and has been the most widely used method for functional assessment in experimental studies [73,74]. However, a comparative study of DPOAEs *versus* brainstem evoked response audiometry in rats that had received systemic cisplatin showed a higher sensitivity of ABR in detecting functional changes attributable to such ototoxicity [75]. 

### 4.2. Auditory Functional Changes on Humans in Clinical Research

Clinical guidelines for ototoxicity monitoring, as the one published by the American Association of Audiology (AAA) (www.audiology.org, October 2009), recommend the use of the following audiological methods to determine a baseline evalutation: pure-tone thresholds in conventional frequency range (PTA), high-frequency audiometry (HFA), tympanometry, speech audiometry and otoacoustic emissions (OAEs). 

Conventional pure-tone audiometric studies (0.25–8 kHz), which is the most extended screening method, show similar results: cisplatin first affects high frequency hearing (above 4 kHz) [76], and then progresses to mid-frequencies (conversational, 500 Hz–3 kHz), when the cumulative dose is greater than 400 mg/m^2^. In patients receiving high-dose cisplatin (150–225 mg/m^2^/dose), audiometric studies of ultra-high frequency demonstrated hearing loss in 100% of patients [77]. While all evidence indicates that cisplatin ototoxicity is dose-dependent and progressive, the largest prospective study evaluating audiometric data from patients treated with cisplatin showed a tendency of hearing loss to reach a plateau, with a clear predominance of the lesion in the higher frequencies: for frequencies above 8 kHz the average hearing loss stood at 75–80 dB, whereas for frequencies below 8 kHz, the loss was placed at around 45–6 dB [78]. As mentioned above, the earliest significant changes in the status of the auditory system due to cisplatin ototoxicity occur at frequencies higher than 8 kHz, which cannot be detected by conventional PTA. Therefore other techniques as HFA or DPOAEs, that can detect auditory changes at an earlier stage of damage, are recommended by AAA for ototoxicity monitoring.

High frequency audiometry is the measurement of pure-tone thresholds at frequencies higher than 8 kHz (9–20 kHz), which can detect changes in the auditory function before ototoxicity affects hearing at conversational frequencies (below 8 kHz) [79]. Knight *et al.* [80] demonstrated that HFA have the potential to reveal earlier changes in auditory function than conventional frequency audiometry during platinum chemotherapy in children, and therefore is a suitable strategy for ototoxicity monitoring. Unexpectantly, this study revealed a trend for ototoxic changes to occur first in HFA thresholds than in DPOAEs, although failed to achieve statistical significance. However, a limitation of the use of this method is the lack of reliability in children under 5 years-old or showing limited attention. Other cases of limitation for the use of HFA, are those patients with hearing loss in the conventional frequency range, which may not have measurable hearing at high frequencies, or have limited responses available for monitoring due to pre-existing losses of OHCs in the cochlear basal region, specially in elder population [81]. For those cases, techniques which do not require the patient’s cooperation will be employed.

The recording of DPOAEs is an objective, sensitive technique that can be used to screen for cisplatin-induced hearing loss, even in pre-symptomatic stages [82], which do not require a behavioral response and are time efficient. One important limitation of OAEs testing is that results may be significantly affected by middle ear pathology such as otitis media [83], which is more common in immunosuppressed chemotherapy patients, as well as in patients receiving head and neck radiation. For this reason, and as mentioned above, guidelines recommend using a test battery, including PTA, HFA, speech audiometry, and tympanometry, besides OAEs.

The testing of auditory evoked potentials (ABR) is another objective and sensitive technique to avaluate hearing loss and ototoxicity monitoring. The possibility of obtaining ABR thresholds at frequencies above the conventional audiogram to monitor ototoxicity has been evaluated [84]. However these methods have yet to obtain widespread clinical use, and high frequency-ABR testing remains in the investigation field [85]. HF-ABR would be most needed in populations who cannot provide reliable behavioral respondes for HFA such as young childred on chemotherapy. However, the use of sedation to obtain reliable ABR recording and track changes over time seems unadvisable and likely contraindicated because of their other health and medication issues.

## 5. Clinical Manifestations of Cisplatin Ototoxicity

Cisplatin-induced ototoxicity lesions usually appear in early stages (from hours to days after exposure) [86], leading to symmetrical [76], progressive, irreversible, cumulative and dose-dependent bilateral sensorineural hearing loss. The effect starts at doses from 60 mg/m^2^ per cycle [87]. In high dose administration schedules, in the 150–225 mg/m^2^/dose range, up to 100% of patients have been found to be affected [77,88]. The hearing loss is associated with tinnitus [89], which affects the majority of patients exposed. Some studies have demonstrated an elevated auditory threshold in 75%–100% of cisplatin-treated patients [90]; among them, pediatric patients were found to be especially susceptible to cisplatin ototoxicity [83,91,92]. 

The reported incidence of cisplatin-related ototoxicity in children ranges from 22% to 70% [91,93,94,95]. Young children are at more risk of developing moderate to severe hearing loss from cisplatin than adults [94,95,96], and there are significant long-term implications, particularly if the children are prelingual or in the early stages of language development [80] or have other functional impairments such as visual deficitis or cognitive dysfunction. This population group do not have the language base or neurologic maturity to fill in the gaps when hearing loss is associated, and therefore they require greater audibility for speech recognition and comprehension [97]. Since high-frequency speech sounds are critical to speech intelligibility, even mild hearing loss in the high frequencies may affect academic and social-emotional development in young children [98,99,100]. Overall, it is considered the most common dose-limiting side effect of this agent [1,3,5,6,7].

Considerable interindividual variability in susceptibility to cisplatin ototoxicity has been described. However, the following are considered risk factors: high cumulative dose of cisplatin, extreme ages of life, renal failure, pre-existing hearing loss, noise exposure [2,101], nutritional deficiency states (including anemia and serum hypoalbuminemia) [88] and radiotherapy affecting the cochlea at doses higher than 48 Gy (most often administered in patients with nasopharyngeal carcinoma) [101,102]. The predicted shift of hearing thresholds in patients treated with radiation therapy and high doses of cisplatin (100 mg/m^2^/dose) is about 10 dB, on average [103]. Particularly in children, cumulative cisplatin dose and male gender were found to be independent risk factors for developing ototixicity [104]. In the cohort study by Yancey *et al.*, where 102 children received cisplatin for the treatment of different neoplasms, it was suggested that for the same cumulative dose of different regimens, the dosing schedule per course may affect toxicity profiles. Those patients receiving 5 days of cisplatin at 20 mg/m^2^/dose (for germ cell tumor treatment) had less hearing loss than those receiving a single day of 100 or 120 mg/m^2^/dose (neuroblastoma and osteosarcoma treatment). In the same study, male gender showed to be more likely to experience hearing loss than female, even after stratifying according to tumor groups (thus, avoiding regimen of dosing schedule becoming a confounding factor) [104]. In agreement with this result, Huang *et al.* [105] concluded that there might exist gender-related genetic differences in cisplatin sensitivity, being male more susceptible for cytotoxicity. They studied the half maximal inhibitory concentration (IC_50_) as an indicator or the effectiveness of four chemotherapeutic agents at inhibiting biological function. Certain cell lines derived from females were less sensitive to platinating agents (including cisplatin) than males when IC_50_ was evaluated.

In addition, several genetic traits predisposing to cisplatin ototoxicity have been described. These include mitochondrial mutations (European mitochondrial haplogroup J, which is very infrequent and is also associated with Leber’s hereditary optic atrophy) [106]; functional polymorphisms of glutathione S-transferase enzyme (four times higher risk of hearing loss in patients exposed to cisplatin with 105Ile/105Ile-GSTP1 or 105Val/105Ile-GSTP1 than with 105Val/105Val-GSTP1 alleles) [107]; non-synonymous single nucleotide polymorphisms (SNPs) of the megalin gene (higher frequency of the A-allele of rs2075252 in individuals with hearing impairment than in individuals with normal hearing after cisplatin therapy) [108]; polymorphisms of the repairing genes excision repair cross-complementing 1, 2, 4 and 5 (ERCC1, ERCC2, ERCC4 and ERCC5), XPA and XPC (greater ototoxicity in patients with the Lys939Gln XPC genotype, in contrast to the greater antitumor response of individuals homozygous for the T allele of the ERCC2 gene) [109].

## 6. Otoprotection strategies

In clinical practice, ototoxicity is the major dose-limiting side effect of cisplatin, requiring discontinuation and replacement by another second-line chemotherapeutic agent, usually carboplatin, or accepting the hearing loss, which is sometimes severe. In order to reduce ototoxicity caused by cisplatin, the potential otoprotective value of many drugs has been studied. The ideal otoprotectant should gather the following properties: it should offer reliable otoprotection, without interfering with antitumoral effect, have minimal adverse effects and use simple administration techniques. The fact that the mechanisms responsible for the tumoricidal action and the ototoxic effect of cisplatin are basically the same is a major obstacle for the use of systemically applied otoprotectants. With the aim of saving this serious limitation, local otoprotection strategies have been developed.

A particular focus has been placed on antioxidants that can mitigate the effects of reactive oxygen and nitrogen species [7]. 

There are three possible strategies to avoid the toxicity caused by reactive oxygen and nitrogen species: preventing ROS interaction with proteins, lipids and cellular DNA by forming complexes with cisplatin that are metabolically inactive; preventing ROS formation; and inducing the production of endogenous antioxidants [110].

### 6.1. Preventing ROS Action

Two molecular mechanisms have been described to inactivate the action of cisplatin: the formation of complexes with cisplatin, which are inactive; and counterion condensation (ion accompanying ionic species to maintain electrical neutrality) [111].

Highly nucleophilic compounds (electron donor agents, which tend to form links with molecular nuclei) react with platinum atoms (Pt), whose nucleus is highly electrophilic (electron-accepting species capable of forming new links), resulting in inactive molecular complexes [110]. Some molecules with nucleophilic properties are those containing sulfur, selenium, carboxylic acid or alcohol groups [111]. Structures containing sulfur atoms are highly nucleophilic, due to the high density of electrons around the nucleus. Therefore, all chemical compounds containing a thiol group (-SH) are assumed to have high affinity to form complexes with cisplatin, neutralizing its activity. Molecules containing a thiol group include amifostine, WR-1065, *N*-acetylcysteine, d-methionine, sodium thiosulfate and erdosteine [111,112]. On theoretical grounds, the selenium atom in ebselen is more nucleophilic than sulfur, due to its greater polarity. However, when ebselen and allopurinol were combined in an *in vivo* model in a study by Lynch *et al.*, the antitumoral activity of cisplatin did not decrease. Therefore, it was concluded that the formation of inactive complexes with cisplatin was limited [113]. Carboxyl and alcohol groups are also nucleophilic, but their potency is limited and they are therefore less efficient at forming inactive complexes [111].

The counterion mechanism consists in the binding of a cationic molecule at physiological pH around the DNA molecule, which has a negative electric charge, so the formation of platinum-DNA adducts is prevented. Some molecules showing counterion properties are WR-1065, allopurinol and ebselen [111].

The most studied drug groups are those containing a thiol group, including sodium thiosulfate, diethyldithiocarbamate, d,l-methionine, methylthiobenzoic acid, lipoic acid, *N*-acetylcysteine, tiopronin, glutathione methyl ester and amifostine, which have demonstrated their otoprotective capacity in experimental studies [109,110,111,112,113,114,115]. However, the use of these drugs has been associated with decreased antineoplastic cisplatin activity, so their clinical utility may be limited [112].

Sodium thiosulfate binds platinum forming inactive complexes [116], and protective actions such as nephroprotection have been demonstrated [117]. However, the antitumor chemotherapy activity is also reduced [118]. Wang *et al.* [114] described a rat model in which cisplatin was administered systemically, while sodium thiosulfate was administered locally with an intracochlear infusion. The histological data demonstrated complete preservation of outer hair cells. The intracochlear perfusion model was too aggressive to be applied to patients, so subsequent studies proposed a less invasive local administration method. Wimmer *et al.* [119] administered topical sodium thiosulfate on the round window of chinchillas that had also received systemic cisplatin. The results did not show otoprotection in otoacoustic emissions. 

Amifostine is an organic thiophosphate compound. Its active metabolite is WR-1065, resulting from amifostine dephosphorylation by alkaline phosphatase in the blood. WR-1065 is a scavenger of oxygen free radicals and prevents the formation of adducts between platinum and DNA [120]. However, in two clinical trials in humans, intravenous administration of amifostine did not show an obvious otoprotectant effect against cisplatin. Two explanations were proposed: amifostine and WR-1065 cannot cross the blood-inner ear barrier; or outer hair cells (the desired target of WR-1065) are too far away from cochlear capillaries and therefore are not exposed to effective concentrations of WR-1065 (which is synthesized in the blood) [121,122].

d-methionine is an amino acid that can prevent the decrease in concentration of endogenous antioxidants (superoxide dismutase, catalase and glutathione) caused by cisplatin in the cochlea [123]. Its mechanism of action involves the formation of d-methionine-cisplatin complexes, thus decreasing the concentration of free cisplatin [122,123,124]. These complexes are known to retain cisplatin’s antitumoral activity [125], and therefore d-methionine may have an otoprotective effect without compromising antineoplastic activity after systemic administration. However, Korver *et al.* [126] avoided the systemic route, and evaluated d-methionine’s utility as an otoprotectant in a chinchilla model administering it on the round window prior to administration of cisplatin also on the round window. Their results demonstrated histological preservation of the outer hair cells and hearing function as evaluated by ABR, compared to animals treated with cisplatin without topical administration of d-methionine [126]. l-methionine showed very similar results to its isomer, both in histological and ABR terms, in a study by Li *et al.* in which the amino acid was administered by mini-pump perfusion on the round window of rats starting before, and continuing throughout, the cisplatin treatment [127].

Erdosteine is a mucoactive compound that contains two sulfur atoms. Although it does not have a free thiol group, a sulfhydryl group is generated by hepatic metabolism to its active metabolites. Kalcioglu *et al.* demonstrated that oral administration of erdosteine in rats for 5 consecutive days after administration of cisplatin preserves the ABR thresholds, compared to animals who did not receive erdosteine [128].

### 6.2. Preventing ROS Formation

Glucocorticosteroids (prednisone, dexamethasone and methylprednisolone) have also been studied for their potential otoprotectant action against cisplatin, based on clinical experience of their use in the systemic treatment of a wide variety of causes of sensorineural hearing loss: inner ear autoimmune processes, endolymphatic hydrops, Ménière’s disease, tinnitus, sudden hearing loss and idiopathic rapidly progressive hearing loss [129,130]. Experimental studies have shown that glucocorticosteroids limit the formation of ROS in the inner ear and have an otoprotective effect against the toxicity caused by aminoglycosides [131]. The presence of corticoid receptors in the inner ear of mice has been demonstrated [130], and some of their actions have been identified. These include electrolytic regulation (regulation of the expression of the Na^+^/K^+^-ATPase and the epithelial sodium channels); their contribution to maintaining the potential in the endolymphatic fluids that bathe the vestibular and auditory hair cells; and immunological control [130]. However, the systemic administration of glucocorticoids as clinical otoprotectors does not appear a useful strategy. Besides their well-known side effects (hyperglycemia, peptic ulcer, hypertension, osteoporosis, *etc.*), they have been shown to reduce the efficacy of antitumor agents by inhibiting apoptotic genes in the tumor cells [130]. 

Allopurinol is a xanthine oxidase inhibitor that can inhibit the formation of ROS. Lynch *et al.* demonstrated the synergic effect of combined administration of allopurinol and ebselen in rats receiving cisplatin [113]. Better preservation of outer hair cells and ABR thresholds was obtained by the combined administration of low doses of both of the compounds than by higher doses of each compound administered separately. A preliminary study by Lynch *et al.* on breast and ovarian cancer models in rats also showed that the combination of allopurinol and ebselen did not decrease the antitumor activity of cisplatin in the breast model, and even improved this activity in the ovarian model [132].

JWH-015 is a ligand of the cannabinoid-2 receptor that was demonstrated to inhibit apoptosis in a dose-dependent manner in an auditory cell line. This is achieved by means of a reduction in ROS production in addition to caspase inhibition, a reduction in mitochondrial release of cytochrome c, and a reduction in production of TNF-α [133].

### 6.3. Inducing the Production of Endogenous Antioxidants

*N*-acetylcysteine (NAC) is an analog of cysteine that has antioxidant activity and induces the synthesis of glutathione, the main endogenous free radical scavenger. Dickey *et al.* demonstrated the otoprotectant effect of NAC in rats by systemic administration prior to systemic cisplatin treatment. Better preservation of hearing in rats pre-treated with NAC than in rats exposed to cisplatin alone was recorded by ABR assessment [112]. Choe *et al.* also demonstrated an otoprotectant role of NAC, preventing ABR alterations, when it was intratympanically injected prior to systemic administration of cisplatin in a chinchilla model [115]. However, recent studies have shown that co-administration of cisplatin and NAC in human tumor cell lines reduces the cytotoxic and apoptotic effects of cisplatin [134]. 

Salicylate is another antioxidant agent that has demonstrated otoprotectant activity. In an experimental rat model, subcutaneous salicylate was administered previous to systemic cisplatin, resulting in lower ABR thresholds and elevated endogenous antioxidants in the cochlea [135,136]. In a similar study by Li *et al.*, systemic co-administration of salicylate in a rat model was found to provide otoprotection without compromising the antineoplastic activity of cisplatin [137]. 

Kalkanis *et al.* found that vitamin E systemically administered prior to cisplatin had otoprotective activity in a rat model [138]. Normal ABR thresholds and preserved outer hair cells were revealed by electron microscopy assessment. More recently, Paksoy *et al.* [139] also described the otoprotective function of vitamin E. They found a reduction in the ABR threshold shift after intratympanic administration of vitamin E in rats previously treated with cisplatin.

d-methionine and l-methionine are capable of preventing the decrease caused by cisplatin in the concentrations of endogenous antioxidants (superoxide dismutase, catalase and glutathione) in the cochlea [123], which might explain its protectant action from noise- and aminoglycoside-induced hearing loss [140]. As mentioned above, another mechanism of action is the formation of methionine-cisplatin complexes, which decreases the concentration of free cisplatin [122,127], without interfering with cisplatin’s antitumoral action [125].

Ebselen can also be cited here as an analog of glutathione peroxidase acting as an antioxidant that inhibits lipid peroxidation without interfering with the antitumor action of cisplatin [113]. Ebselen’s otoprotectant effect against cisplatin has been demonstrated in rats by Rybak *et al.* [141].

According to Whitworth *et al.* [64], adenosine agonists, including R-phenylisopropyl adenosine (R-PIA) and 2-chloro-*N*-cyclopentyl adenosine (CCPA) administered on the round window of chinchillas before cisplatin treatment also on the round window, ameliorated the preservation of ABR thresholds, reduced cell damage and reduced lipid peroxidation. Cisplatin stimulates the synthesis of A1 adenosine receptors in the cochlea. This is part of the natural antioxidant defensive system of the cochlea [142]: when this receptor is activated by adenosine agonists, the cochlear concentrations of endogenous antioxidants are increased, providing protection against oxidative stress [143].

### 6.4. Other Mechanisms

In the Choe *et al.* study mentioned above [115], lactate (contained in the Ringer solution initially used as vehicle) was demonstrated to have even greater otoprotective potential than NAC, when both were administered intratympanically in chinchillas that later received systemic cisplatin. The low molecular weight of lactate (89.1 Da compared to 163.2 Da NAC) could explain its greater permeability through the round window [115]. The mechanism of action accounting for lactate’s otoprotective effect is still unknown [7].

Melanocortins are neuropeptides derived from adrenocorticotropic hormone (ACTH) that have neuroprotective properties such as acceleration of recovery and induction of peripheral nerve regeneration [144]. Specifically, Org2766, a synthetic ACTH (4-8) analog, has been studied as a neuroprotective agent against cisplatin. Van der Hoop *et al.* [144] showed that systemically administered Org2766 in patients with advanced ovarian cancer treated with cisplatin had a neuroprotective action, with no decrease in antitumor activity. However, it has not yet been demonstrated whether this neuroprotection also includes the outer hair cells. One animal study focused on the otoprotector effect of Org2766 in coadministration with cisplatin. However, the preservation of cell counts was only partial and interindividual variability was too high for a firm conclusion [145].

Some neurotrophins, such as neurotrophin-3 (NT-3) and brain-derived nerve growth factor (BDNF) have also been proven to be effective as otoprotectors against cisplatin [146].

A purified extract of *Ginkgo biloba* also showed a protective effect on outer hair cells and prevented ABR threshold elevation in rats receiving cisplatin [147]. 

Pifithrin-α, a p53 inhibitor agent, had a cytoprotective action on outer hair cells *in vitro*, blocking caspase activation and subsequent apoptosis [148].

Research has also been carried out on gene therapy, with an adeno-associated viral vector encoding the X-linked inhibitor of apoptosis (XIAP), administered on the round window before cisplatin administration through the same route. Despite the favorable results in terms of a reduction in cell death and preservation of ABR thresholds, its potential use in clinical practice was dismissed due to the aggressiveness of the process (the approach to the round window) and the inflammatory process triggered in the inner ear by the viral vector [149].

## 7. Local Administration of Otoprotective Drugs

As discussed above, the clinical use of otoprotector substances administered by systemic routes is limited by their side effects, and especially by the resulting reduction in the efficiency of the antitumor treatment [3,6,130]. Consequently, numerous clinical and experimental studies have explored the possibility of using a local route to administer otoprotector drugs. The local route of administration allows much higher concentrations of the drug to be achieved in the inner ear than elsewhere in the body, in comparison to oral or parenteral routes, thus avoiding systemic side effects and decreased tumor efficacy [3,6,129,131,150]. Several animal models have demonstrated the otoprotectant role of different drugs administered locally [64,115,119,126]. In most animal models, drug administration has been accomplished by opening the temporal bulla by means of a retroauricular surgical approach under general anesthesia. The drug is then administered right on the round window, by direct viewing of the structures. The cisplatin toxicity model used in many of these studies was local administration of the chemotherapeutic agent on the round window of both ears, also using a retroauricular surgical approach, a few minutes (30–90 min) after the administration of the otoprotectant in one of the subject’s ears, using the contralateral ear as a control [64,126]. The toxic effect of cisplatin on the cochlea by its local administration on the round window has been described in several murine models [151,152,153]. A less-used technique is transtympanic puncture and drug instillation, filling the entire volume of the middle ear. It is assumed that the drug will contact the round window, although this technique does not allow direct viewing of this structure [152,154].

In clinical practice, transtympanic or intratympanic (IT) puncture is the technique of choice for local administration, as the surgical approach needed to reach the inner ear would be far too aggressive. Drugs that are intratympanically administrated are supposed to enter the inner ear (tympanic ramp) through the round window membrane. This membrane is a three-layered semi-permeable structure: the outermost surface (that exposed to the middle ear) is covered by an epithelium of cuboidal cells joined by tight junctions; the surface exposed to the inner ear is covered by mesothelial cells often in an intermittent arrangement; and between them there is a network of collagen fibers, elastic fibers, fibrocytes, fibroblasts, nerve fibers and vessels. Despite the existence of intercellular junctions in its outermost surface, molecules up to 1 micrometer in diameter can easily penetrate the membrane reaching the inner ear either intercellularly or transcellularly by pinocytosis [129]. Structurally, the human round window is very similar to that of the animal species used in experimental studies; the only relevant difference is the thickness: 60–70 microns in human *versus* 10–14 microns in rodents (rat, chinchilla). Therefore, the main difference between species is the time it takes the molecule to pass through the fibrous layer [150,155]: thinner membranes offer greater permeability [156]. The communication between the scala tympani and both the organ of Corti and the spiral ganglion exposes hair cells and nerve cells to substances administered IT [156]. Several studies have shown the existence of a decreasing concentration gradient of substances from the base to the apex of the cochlea, after their IT administration [130,150,155,156].

The physico-chemical properties of drugs administered IT also determine their capacity to permeate into the inner ear. Their molecular weight, electric charge and lipophilicity play an important role. Models both *in vitro* and *in vivo* showed that substances with a low molecular weight can cross the round window much more readily than those with a high molecular weight [157].

For the same molecule, the variability of IT therapeutic results may be due to interindividual differences, such as: the presence in front of the round window of mucous adhesions or false membranes that have a shielding effect; variations in the permeability of the round window; and the emergence of drug concentration gradients in different regions of the inner ear [130,156]. With the aim of reducing this therapeutic variability, several studies have addressed the question of improving the vehicles that carry the effective drugs. These vehicles are biodegradable molecules (fibrin glue, hyaluronic acid, and poly-lactic/glycolic acid [PLGA]) that afford sustained release of the drug, providing stable concentrations in the inner ear after the administration of the drug-vehicle mixture directly on the round window [114,158,159].

Our understanding of the IT route may be less satisfactory than presumed. Thus, recent experimental studies contradict the widely accepted theory that defines the round window as the entry point from the middle ear to the inner ear, and posit that the structure that allows access to the inner ear is the oval window. Two murine models of gadolinium (Gd) distribution into the inner ear after administration on different structures of the middle ear showed that the oval window is a much more effective route of entry than the round window. The crossing pathway would be the annular ligament of the stapedius-vestibular joint [160,161]. A similar conclusion was obtained in an animal study that evaluated histological and functional injury after local administration of a toxicant (gentamycin). The results showed that the lesion was much more severe (both in the cochlea and the vestibule) when the drug was administrated on the oval window than when it was administrated on the round window [162].

The most widely studied group of otoprotector drugs for local administration is the glucocorticoids, due to extensive clinical experience in their use. The first recorded case of intratympanic corticosteroid administration was performed in 1956 by Schuknecht to treat Meniere’s disease [163]. Since then, glucocorticoids have been used to treat sudden sensorineural hearing loss, tinnitus, autoimmune inner ear disease and Meniere’s disease. Experimental models have also described the otoprotector activity of local administration of glucocorticoids against the toxicity caused by cisplatin. Intratympanic administration of dexamethasone has demonstrated otoprotectant action against cisplatin both in terms of structural and functional parameters (DPOAEs, ABR), without evidence of associated ototoxic or systemic side effects in several rodent models: mouse [3,164], rat [6,165] and guinea pig [142,166,167]. One of these studies evaluated the chronological relationship between the administration of the otoprotectant and the chemotherapeutic agents, and found that the otoprotectant potential action of intratympanic dexamethasone is only effective if administered at a time close to the cisplatin administration. Precisely, it was effective when administered one hour before cisplatin, but ineffective when administered one day prior to cisplatin [167]. Recently Hughes *et al.* [168] reported an experimental study in mice in which no statistically significant differences were observed between ABR thresholds of the ear injected with dexamethasone and the contralateral ear (injected with saline) in individuals previously treated with cisplatin. The authors attributed these findings to the possibility that the corticosteroid had reached the contralateral ear, exerting partial otoprotection there. Although this hypothesis is supported by some authors [166,169], others reject it arguing that the solutions given in the control ears (saline, lactate) have shown some otoprotective actions that could explain these effects [6,115], and that extensive clinical experience with unilateral transtympanic injection of ototoxic substances (gentamicin) have not resulted in evidence of contralateral audiometric loss or vestibular dysfunction in the patients [170].

Although less commonly used, intratympanic methylprednisolone has also been demonstrated to be otoprotective against cisplatin in guinea pigs [171]. A study on guinea pigs comparing the pharmacokinetic behavior of three glucocorticoids (dexamethasone, hydrocortisone and methylprednisolone) administered IT, showed that methylprednisolone reaches higher concentrations in the inner ear and for a longer duration than dexamethasone and hydrocortisone [130]. However, this study did not evaluate the potential otoprotector effect of these drugs.

## 8. Clinical Studies with Otoprotective Drugs

As reviewed above, numerous animal and *in vitro* studies support the use of protective agents to mitigate hearing loss caused by cisplatin. However, human data are scarce and no useful prophylactic treatment has reached consensus for widespread clinical application [172]. A search of the US NIH clinical trials database (www.clinicaltrials.gov) on 24 March 2015 revealed several trials testing the efficacy of otoprotective treatments against cisplatin. Tested agents included sodium thiosulfate, amifostine, *N*-acetylcysteine, salicylate, ebselen, lactate, dexamethasone, and Ginko biloba extract. In several of these studies, the route of administration was IT. Published literature is scarce. Nevertheless, Riga *et al.* reported positive otoprotection evidence for IT *N*-acetylcysteine [173] by comparing auditory thresholds in the treated and contralateral (untreated control) ears of the same patients who had received cisplatin. Although previous clinical studies failed to demonstrate an obvious otoprotectant effect of amifostine [121,122], a recent study reported that systemic administration of this drug is otoprotective in average-risk, but not high-risk, children treated with cisplatin for medulloblastoma [174].

## 9. Conclusions

Ototoxicity is a serious side effect of cisplatin. The cellular and molecular bases of this effect are fairly well—but not completely—understood at present. Critical steps in cisplatin ototoxicity are the entrance of the drug and its toxic metabolite cisdiaminne(aqua)chloroplatinum into the sensory hair cells, and the generation in the cell of oxidative stress and DNA damage finally triggering apoptosis. On the basis of this knowledge and clinical experience, a range of protective treatments have been proposed and many of them have been found to be active in cell culture and animal models. However, scarce data have been available to support clinical interventions that may help to prevent hearing loss in patients treated with cisplatin. Nevertheless, the existence of many clinical trials currently addressing this question suggests that useful preventive treatments will soon be identified. In fact, some positive data are already available [173,174]. In this battle, the accessibility of the auditory system by the IT route widens the possible choices by allowing the use of agents that could not be used by systemic administration because of their excessive toxicity or because they diminish the oncolytic efficacy of cisplatin. 
